# Healthcare-Associated *Clostridioides difficile* Infection: A Hospital-Based Retrospective Study in North Eastern Romania

**DOI:** 10.3390/microorganisms13061377

**Published:** 2025-06-13

**Authors:** Lidia Oana Stămăteanu, Ionela Larisa Miftode, Claudia Elena Pleşca, Mihnea Eudoxiu Hurmuzache, Doina Carmen Manciuc, Daniela Leca, Egidia Gabriela Miftode

**Affiliations:** 1 Doctoral School, University of Medicine and Pharmacy Gr. T. Popa, 700115 Iași, Romania; oana_iavny@yahoo.co.uk; 2 “St. Chiriachi” County Emergency Hospital, 730006 Vaslui, Romania; 3 Department of Internal Medicine II, Faculty of Medicine, University of Medicine and Pharmacy Gr. T. Popa, 700115 Iași, Romania; claudia-elena.badarau@umfiasi.ro (C.E.P.); mihnea.hurmuzache@umfiasi.ro (M.E.H.); doina.manciuc@umfiasi.ro (D.C.M.); daniela.leca@umfiasi.ro (D.L.); egidia.miftode@umfiasi.ro (E.G.M.); 4 “St. Parascheva” Clinical Hospital of Infectious Diseases, 700116 Iași, Romania

**Keywords:** *Clostridioides difficile* infection, healthcare-associated infection, community-acquired, recurrence, comorbidities, mortality, outcomes, risk factors

## Abstract

*Clostridioides difficile* infection (CDI), the most common cause of nosocomial diarrhea, presents with a wide spectrum of clinical manifestations, ranging from mild diarrhea to severe, life-threatening conditions such as pseudomembranous colitis and toxic megacolon. In recent years, both the incidence and severity of CDI have increased, leading to a significant burden in terms of morbidity, mortality, and healthcare costs. We conducted a single-center, retrospective cohort study for 30 months at “Sf. Parascheva” Infectious Diseases Clinical Hospital Iași, in North Eastern Romania, aiming to assess the clinical and laboratory characteristics of CDI, as well as treatment approaches and their association with patient outcomes. A total of 534 patients were included during the study period, of whom 484 had favorable outcomes, while 50 have died of the disease. Fever (*p* = 0.007) and age over 65 (*p* = 0.001) were associated with prolonged hospitalization. Patients positive for both A and B toxins and GDH had the highest risk of recurrence (*p* = 0.020). Among comorbidities, obesity was the only condition significantly linked to recurrence (*p* = 0.001). In female patients over 65 years old, the probability of survival drops below 60% after 21 days of hospitalization, highlighting a critical risk factor in this population. These results underscore the importance of comprehensive risk assessment in CDI, particularly focusing on advanced age and comorbidities, to guide early therapeutic interventions, optimize patient management, and improve clinical outcomes among high-risk populations.

## 1. Introduction

*Clostridioides difficile* (previously known as *Clostridium difficile*) (CD) is a leading cause of antibiotic-associated diarrhea and is responsible for the majority of pseudomembranous colitis cases following antibiotic therapy [1]. Although historically identified as a pathogen primarily responsible for healthcare-associated infections, CD is now increasingly prevalent in community settings, causing disease in individuals without any predisposing risk factors [2].

It is well established that the primary risk factors for *Clostridioides difficile* infection (CDI) include antibiotic use, hospitalization, advanced age, and proton pump inhibitor therapy [3,4,5]. In cases of community-acquired CDI, key risk factors involve contamination of water and food, the presence of epidemic strains, and asymptomatic carrier status [6,7,8,9]. The most common antibiotic classes associated with CDI are lincosamide (clindamycin), cephalosporins, fluoroquinolones, and carbapenems. Other classes, such as tetracyclines, sulfonamides, and sulfamethoxazole-trimethoprim, are also involved, though with lower incidence rates of CDI [4,10,11,12,13].

The clinical manifestations of CDI range from asymptomatic colonization to severe conditions such as pseudomembranous colitis, toxic megacolon, sepsis, and potentially fatal outcomes [2,14,15].

CDI is a toxin-mediated disease primarily determined by the production of toxin A (TcdA) and toxin B (TcdB), which are encoded by the tcdA and tcdB genes, which are part of the CdtLoc locus [6,16,17].

According to the European Society for Clinical Microbiology and Infectious Diseases (ESCMID), the optimal approach to CDI diagnosis is to perform a two-step algorithm. Initially, a screening test with high sensitivity and negative predictive value, such as a standalone nucleic acid amplification test (NAAT) or a glutamate dehydrogenase (GDH) test, should be performed. If the result is positive, it must be followed by a more specific test with a high positive predictive value, such as an enzyme-linked immunoassay (ELISA), to detect free toxins and confirm the diagnosis. Alternatively, if the second test is negative patients should undergo clinical evaluation, as they may either have an active infection with toxin levels below the detection threshold or may be carriers of a toxigenic strain [18,19].

According to the latest guidelines, CDI can be treated with oral vancomycin (125 mg four times daily) or fidaxomicin (200 mg twice daily) in non-severe cases [20]. Although metronidazole has been used as a therapeutic option for non-severe cases, evidence now confirms that oral vancomycin is more effective [21,22]. In severe or fulminant cases, the vancomycin dose can be increased to 500 mg four times daily, and intravenous metronidazole (500 mg three times daily) may be added as an adjunct therapy [20]. Fecal microbiota transplantation (FMT) is recognized as a highly effective therapy for recurrent CDI [20,23,24,25,26,27]. This procedure involves the transfer of fecal matter from a healthy donor into the recipient’s gastrointestinal tract, with the goal of restoring a balanced and functional intestinal microbiome [26,27].

The evolution of CDI is marked by an increase in mortality rates among elderly patients, particularly when the infection manifests as a severe form [13,28]. A further concern is the high rate of recurrence, which significantly increases the possibility of additional episodes following an initial relapse [2,4,29].

The aim of this study was to identify clinical, epidemiological, and therapeutic factors associated with disease severity, recurrence, and mortality in patients diagnosed with CDI. We evaluated the impact of patient characteristics, comorbidities, toxin profiles, and treatment regimens on hospitalization duration, clinical outcomes, and infection recurrence. Additionally, we analyzed specific risk factors for nosocomial versus community-acquired CDI, as well as predictors of unfavorable outcomes in elderly patients, in order to provide insights into optimizing patient management and preventing adverse outcomes.

## 2. Materials and Methods

We conducted a single-center observational and retrospective study using patients diagnosed with CDI between January 2016 and June 2018 in “Sf. Parascheva” Infectious Diseases Clinical Hospital Iași, in North Eastern Romania. All participants in the study were adult (≥18 years), hospitalized at our hospital, and had a confirmed diagnosis of CDI.

**Figure 1:**
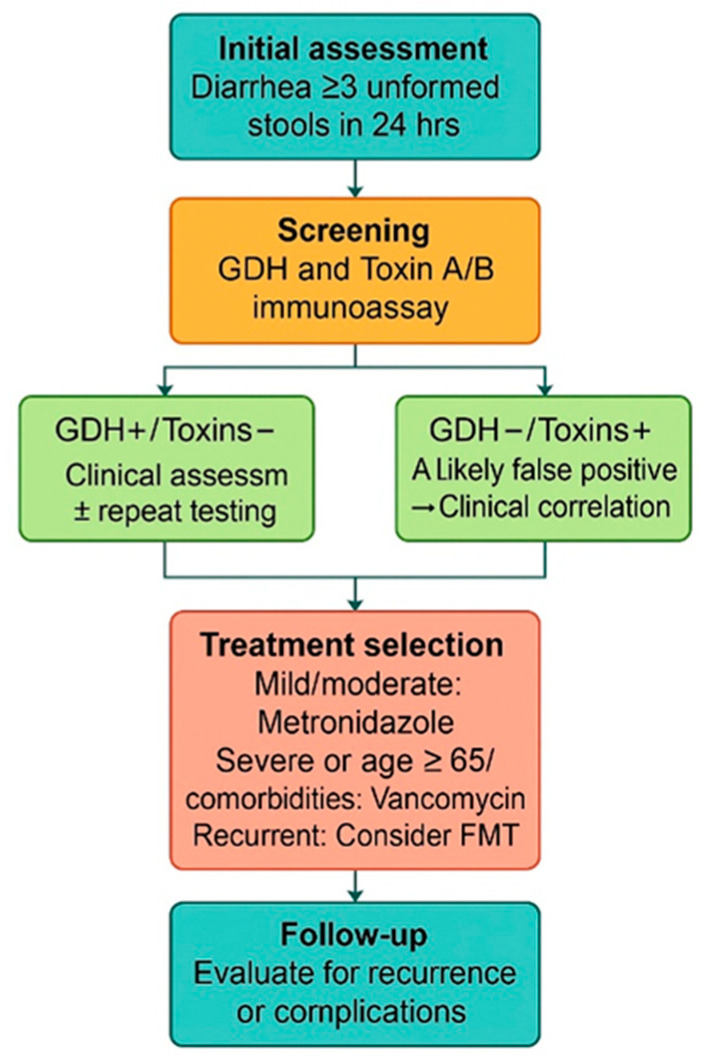
Diagnostic and therapeutic pathway for patients with CDI included in the study.

A confirmed diagnosis of CDI was characterized by diarrhea, defined as the passage of three or more unformed stools within a 24 h period, along with a positive test for CD toxins A and B (by chromatographic immunoassay qualitative testing), as well as GDH antigen. Detection was performed using the CerTest C. difficile GDH + Toxin A/B Combo Test assay (CerTest Biotec, Zaragoza, Spain), a rapid chromatographic immunoassay that employs monoclonal antibodies to simultaneously identify glutamate dehydrogenase (GDH) and toxins A and B. This method is widely utilized in clinical microbiology laboratories for initial screening and diagnostic confirmation (Figure 1). The assay provides individual results for each toxin, enabling the separate detection of toxin A and toxin B.

A total of 534 patients were included during the study period. Patient information was collected from the patients’ medical records, including sociodemographic information (age, gender, and place of origin), comorbidities (such as cardiovascular diseases, diabetes, renal dysfunction, obesity, malignancies, etc.), laboratory findings, as well as clinical and therapeutic data.

The database was compiled and processed in SPSS (IBM Corp. Released 2022. IBM SPSS Statistics for Windows, Version 29.0. Armonk, NY, USA: IBM Corp). Apart from descriptive statistical analysis, univariate multivariate logistic regression tests were performed to compare results and obtain the corresponding *p* values. Statistical significance was defined as a *p* value of less than 0.05.

The study was formally approved by the institution’s research ethics committee and patients provided written consent on admission for their anonymized data to be subsequently used for scientific research purposes.

The objective of this analysis was to perform a comparative assessment, evaluating the alignment between the epidemiological characteristics reported in the existing literature and the findings from our study.

## 3. Results

Between January 2016 and June 2018, 534 patients were diagnosed with CDI and we obtained the information from their personal medical files.

With regard to symptoms and epidemiological characteristics and their impact on length of hospitalization, it was observed that patients presenting with a fever and those over the age of 65 experienced significantly longer hospital stays (Table 1).

We analyzed the correlations between toxin type, comorbidities, and laboratory parameters, finding that patients with gastrointestinal, cardiovascular, and renal comorbidities, as well as those with elevated transaminase levels, showed stronger associations with the presence of toxin A and GDH (Table 2).

We aimed to determine the correlations between toxin profiles and clinical outcomes in our patients. Our analysis revealed that those with toxins A + B + GDH had the highest recurrence rate (30.3%, *p* = 0.020), suggesting a more virulent or persistent infection pattern in this subgroup (Table 3).

Our next analysis suggested that metronidazole treatment in CDI is significantly associated with a lower recurrence rate (*p* = 0.001), while it also showed a trend toward improved outcomes and reduced mortality, although these associations were borderline significant (*p* = 0.050). On the contrary, patients with CDI treated with vancomycin had a lower favorable outcome rate (88.3% vs. 95.8%, *p* = 0.004) and a higher recurrence rate (36.0% vs. 9.1%, *p* = 0.001) compared to those not receiving vancomycin. Mortality was also significantly higher in the vancomycin group (11.7% vs. 4.2%, *p* = 0.004) (Table 4).

Table 5 summarizes the clinical outcomes (favorable evolution, recurrence, and mortality) and treatment approaches in relation to the detected C. difficile toxin profiles (toxin A, toxin B, and toxin A + B). Patients with toxins A + B had the highest recurrence rate (30.3%) and mortality (10.7%), compared to those with toxin A or B alone. Treatment choices varied across groups, with vancomycin being more commonly used in patients with more severe presentations. For contextual comparison, the table also includes ESCMID 2014 guideline recommendations, which guided treatment during the study period. Notably, while ESCMID recommendations are based on disease severity rather than toxin profile, our data suggest that toxin A + B infections may be associated with more severe outcomes.

To provide a more detailed comparison of the enrolled patients and better understand the factors associated with CDI recurrence, we analyzed the clinical and demographic characteristics of the three treatment groups: metronidazole (n = 165), vancomycin (n = 289), and vancomycin combined with metronidazole (n = 80). Significant differences were observed between treatment groups regarding age and length of hospitalization. Patients treated with metronidazole were younger (55 ± 16.2 years) and had shorter hospital stays (7.9 ± 4.3 days) compared to those receiving vancomycin (61 ± 15.8 years; 12.3 ± 6.8 days) or vancomycin + metronidazole (61 ± 14.9 years; 12.0 ± 6.5 days), with both differences being statistically significant (*p* < 0.001) (Table 6).

Abdominal pain was more frequent in the metronidazole (80.6%) and combination (77.5%) groups than in the vancomycin group (68.5%) (*p* = 0.013). Cardiovascular comorbidities were significantly more prevalent among patients receiving vancomycin (31.5%) compared to the other two groups (*p* < 0.001). Obesity was also more common in the vancomycin group (10.0%) than in the metronidazole (3.0%) or combination group (7.5%) (*p* = 0.019) (Table 7).

In the univariate analysis of CDI recurrence, fever (*p* = 0.014) and abdominal pain (*p* = 0.001) were significantly associated with recurrence. Watery stools were present in all cases but lacked statistical significance (*p* = 0.723), while vomiting showed no significant correlation (*p* = 0.353). The presence of toxin B (*p* = 0.029) and toxins A + B (*p* = 0.005) were also significantly associated with recurrence. In the univariate analysis of CDI recurrence and comorbidities, obesity was the only condition significantly associated with recurrence (*p* = 0.041) (Table S1: see Supplementary Materials).

We aimed to identify factors associated with unfavorable outcomes in patients over 65 years old by performing a logistic regression analysis to evaluate potential correlations between disease prognosis and specific comorbidities (pulmonary, renal, and diabetes). In the initial model, the presence of pulmonary comorbidities was significantly associated with unfavorable outcomes, showing a 5-fold increased risk of mortality (OR = 5.344; IC95: 3.664–7.795; *p* = 0.001). In a subsequent model, adding renal comorbidities alongside pulmonary comorbidities also demonstrated a significant association with mortality, with a 2.2-fold increased risk (OR = 2.200; IC95: 21.194–4.053; *p* = 0.001). Lastly, a third model incorporating diabetes (*p* = 0.436) alongside pulmonary (*p* = 0.001) and renal comorbidities (*p* = 0.011) did not show a significant additional increase in mortality risk in this age group (OR = 1.349; IC95: 0.636–2.860; *p* = 0.436) (Table 8).

**Figure 2:**
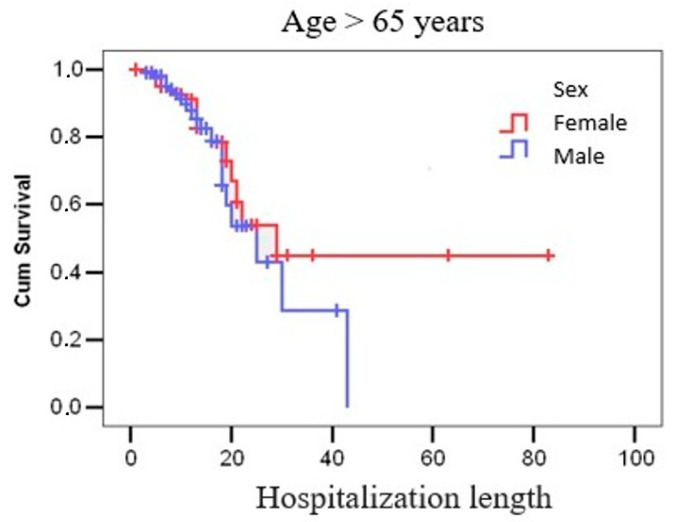
Kaplan–Meier survival curves by sex and age group.

We completed a survival analysis using Kaplan–Meier curves to evaluate the potential correlation between length of hospitalization and mortality in patients over 65 years old. The analysis revealed that in females over 65 years old, the probability of survival decreases to below 60% after 21 days of hospitalization, while in males over 65 years old, survival probability declines to approximately 43% after 25 days of hospitalization (Figure 2).

We conducted an analysis to evaluate the factors associated with nosocomial and community-acquired CDI. Older age (*p* = 0.036), the presence of watery stools (*p* = 0.033), detection of toxin B (*p* = 0.041), and gastrointestinal comorbidities (*p* = 0.044) were all significantly associated with a higher risk of nosocomial CDI. In contrast, vomiting (*p* = 0.001) and cardiovascular comorbidities (*p* = 0.037) were significantly associated with community-acquired CDI (Table S2—see Supplementary Materials).

**Figure 3:**
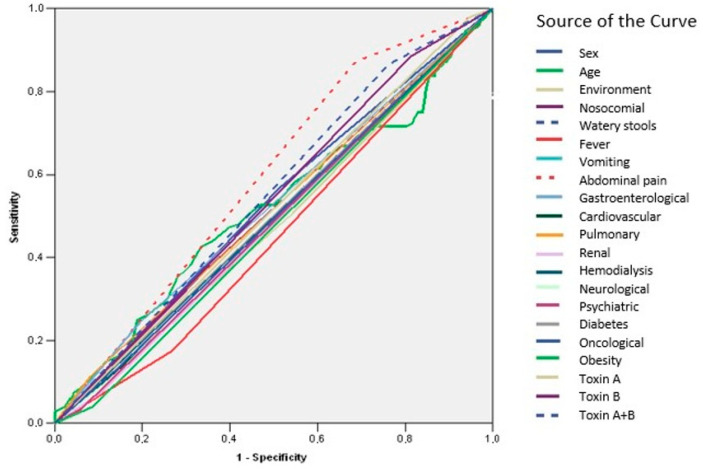
ROC curve—predictors of recurrence.

**Figure 4:**
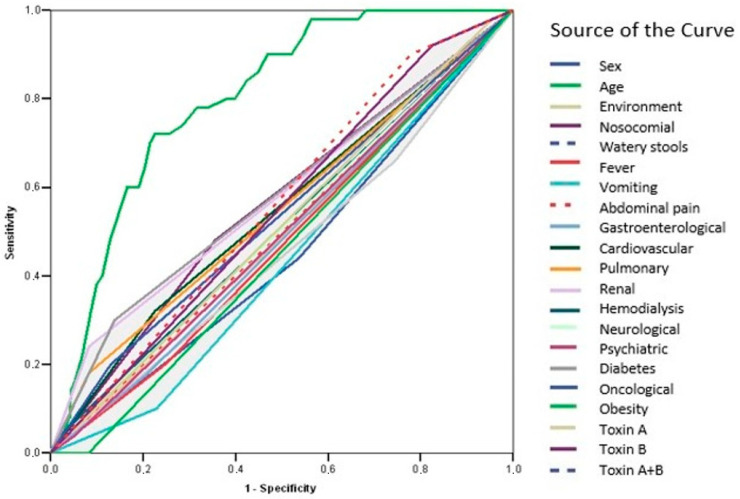
ROC curve—predictors of mortality.

ROC curve analysis revealed that none of the evaluated parameters demonstrated adequate predictive value for recurrence (AUC < 0.700) (Figure 3, Table 9). In contrast, age was identified as a significant predictor of mortality (AUC = 0.798; IC95%: 0.745–0.852; *p* = 0.001) (Figure 4, Table 10).

## 4. Discussion

CDI is an infectious condition resulting from an overgrowth of toxin-producing CD, leading to disruption of the intestinal microbiota. The disease is characterized by intestinal inflammation, accompanied by pseudomembrane formation and toxin-mediated tissue damage [28,30]. Given the global burden of CDI, both in terms of economic impact and associated mortality, advancing research and identifying new therapeutic strategies remain crucial. In this context, we aimed to analyze specific clinical and demographic characteristics of patients diagnosed with CDI and hospitalized in an infectious diseases unit.

In our study we found out that patients presenting with fever (*p* = 0.007) and those over the age of 65 (*p* = 0.001) experienced significantly longer hospital stays. Similar data was found in other studies [31,32]. Fever is not typically observed in common forms of CDI, suggesting that its presence may indicate an associated infection, superinfection, or a more severe CDI presentation characterized by febrile syndrome [33,34].

Analyzing the correlation between toxin type, comorbidities, and laboratory parameters, we found that patients with gastrointestinal, cardiovascular, and renal comorbidities, as well as those with elevated transaminase levels, showed stronger associations with the presence of toxin A and GDH. These observations align with existing literature indicating that certain comorbid conditions can influence the severity and presentation of CDI, and also suggest that the presence of toxin A and GDH are linked to more severe manifestations of the disease, particularly in patients with the aforementioned comorbidities [17,35]. While our findings suggest potential associations between the presence of toxin A and GDH with advanced presentation of CDI, we acknowledge that current evidence strongly supports toxin B as the primary virulence factor in disease severity. The presence of GDH and either toxin generally indicates active infection, but clinical severity is more reliably predicted by patient-specific factors—such as comorbidities, inflammatory markers—and particularly by the presence of toxin B [36,37,38,39]. Many patients in our cohort who were positive only for toxin A exhibited clinically significant symptoms, suggesting potentially false-negative toxin B results, mixed toxin profiles, or less well-characterized virulent strains. These findings highlight the need for further molecular characterization and ribotyping to clarify the toxin gene profiles and their clinical relevance in our setting.

Our analysis aimed to determine the correlation between toxin profiles and clinical outcomes in our patients. We observed that individuals positive for both toxin A and toxin B, along with GDH, exhibited the highest recurrence rates (*p* = 0.020), suggesting a more virulent or persistent infection pattern in this subgroup. This finding aligns with the existing literature indicating that strains producing both toxins A and B are associated with increased disease severity and recurrence. Notably, the hypervirulent BI/NAP1/027 strain, characterized by elevated production of both toxins and the presence of binary toxin, has been linked to higher recurrence rates and more severe clinical outcomes. Among these factors, studies have identified advanced age, gastric acid suppression, prolonged hospitalization, and prior antibiotic use as significant contributors to CDI recurrence [3,29,40,41].

Our results suggest that metronidazole-treatment CDI is significantly associated with a lower recurrence rate (*p* = 0.001). Additionally, there was a trend toward improved outcomes and reduced mortality with metronidazole; however, these associations were borderline significant (*p* = 0.050). Conversely, patients treated with vancomycin had a lower favorable outcome rate (*p* = 0.004) and a higher recurrence rate (*p* = 0.001) compared to those not receiving vancomycin. Mortality was also significantly higher in the vancomycin group (*p* = 0.004). These findings appear to contrast with the existing literature, which often reports comparable or superior outcomes with vancomycin, especially in severe CDI cases [22,42,43]. For instance, a study found that vancomycin was more effective than metronidazole in treating severe CDI, while both antibiotics were similarly effective for mild cases [43]. Another study reported that vancomycin significantly reduced the risk of 30-day mortality in severe CDI cases compared to metronidazole, although recurrence rates were similar between the two treatments [44]. The discrepancy between our findings and those of previous studies may be attributed to differences in study design, patient populations, or definitions of disease severity. Furthermore, in accordance with the treatment protocol at our hospital, metronidazole was reserved for patients with mild forms of CDI, which likely explains the lower recurrence rates observed in this group. This selection bias toward less severe cases may account for the differences between our findings and those of previous studies, where vancomycin is typically associated with a lower risk of recurrence [6,20,21,45].

The clinical outcomes and treatment strategies varied according to the detected CD toxin profile (A, B, and A + B). Among the 534 patients analyzed, those with toxin A + B had the highest recurrence rate at 30.3%, significantly greater than the toxin A (19.1%) and toxin B (13.0%) groups (*p* = 0.020). Mortality was also higher in the toxin A + B group (10.7%) compared to toxin A (4.5%) and toxin B (4.3%), although these differences did not reach statistical significance (*p* = 0.097). Favorable outcomes were observed in over 89% of patients across all groups, without statistically significant differences (*p* = 0.097). Treatment regimens primarily involved vancomycin, consistent with disease severity, with fecal microbiota transplantation (FMT) used infrequently and without significant differences between groups (*p* = 0.582). For reference, the ESCMID 2014 guidelines—which focus treatment recommendations based on disease severity rather than toxin profile—are included for contextual comparison. Our findings suggest that infections with toxin A + B may be associated with more severe clinical evolution and higher recurrence, underscoring the importance of tailored management strategies in this population [25]. These findings are consistent with previous reports indicating that infections caused by strains producing both toxin A and toxin B are associated with more severe clinical outcomes and higher recurrence rates [46,47,48].

The comparative analysis of patient characteristics by antibiotic therapy revealed statistically significant differences, particularly in age, length of hospitalization, and certain comorbidities. Patients treated with vancomycin or the combination of vancomycin and metronidazole were older and had longer hospital stays compared to those receiving metronidazole alone. These findings may reflect more severe clinical forms or a higher burden of underlying conditions in these groups. In particular, cardiovascular comorbidities and obesity were significantly more frequent in the vancomycin group (*p* < 0.001 and *p* = 0.019, respectively), both conditions that may complicate clinical evolution [41,49,50,51,52].

Additionally, the presence of abdominal pain differed significantly between the groups (*p* = 0.013), suggesting a variation in clinical presentation that may have influenced therapeutic decision-making. These differences highlight the heterogeneity of patient profiles depending on antibiotic regimen and may partly explain the therapeutic choices made. It is also important to mention that fidaxomicin was not included in this analysis, as it was neither available nor used in our hospital during the study period. Therefore, no patients received fidaxomicin, and the comparative data refer only to the antibiotic therapies actually administered.

In our univariate analysis of CDI recurrence, fever (*p* = 0.014), abdominal pain (*p* = 0.001), toxin B (*p* = 0.029), the combination of toxins A and B (*p* = 0.005), and obesity (*p* = 0.041) were all significantly associated with an increased risk of recurrence. These findings are consistent with the existing literature that identifies multiple factors influencing CDI recurrence. Advanced age, additional antibiotic use during follow-up, proton-pump inhibitor use, and renal insufficiency have been frequently cited as independent risk factors for recurrent CDI. Moreover, the presence of multiple comorbidities has been associated with an increased risk of severe CDI and recurrence [2,3,29,53].

We conducted a logistic regression analysis to identify factors associated with unfavorable outcomes in patients over 65 years old. The initial model revealed that pulmonary comorbidities were significantly associated with a 5-fold increased risk of mortality. When renal comorbidities were added to the model, the combined presence of pulmonary and renal conditions demonstrated a 2.2-fold increased risk of mortality. However, incorporating diabetes into the model did not show a significant additional increase in mortality risk within this age group. These findings align with the existing literature emphasizing the impact of certain comorbidities on CDI outcomes in older adults. Advanced age is a well-established risk factor for severe CDI and increased mortality. Studies have shown that mortality due to CDI rises from 5% in individuals aged 61–70 years to over 10% in those over 80 years old [54,55]. Additionally, underlying medical conditions, including renal failure, have been associated with higher mortality and complications in CDI patients [56]. Our analysis further underscores the significance of pulmonary and renal comorbidities as predictors of poor outcomes in elderly CDI patients. Interestingly, while diabetes is often considered a risk factor for various infections, it did not show a significant impact on mortality in our cohort [57,58,59]. This suggests that the presence of pulmonary and renal diseases may play a more critical role in influencing CDI prognosis among the elderly.

We conducted a Kaplan–Meier survival analysis to examine the relationship between hospitalization duration and mortality in patients over 65 years old. The results indicated a notable decline in survival probabilities with extended hospital stays. Specifically, for females over 65, the probability of survival decreased to below 60% after 21 days of hospitalization. In males over 65, survival probability declined to approximately 43% after 25 days of hospitalization. These findings highlight the impact of prolonged hospitalization on survival outcomes in the elderly population. The existing literature supports the association between extended hospital stays and increased mortality risk among older adults. For instance, a study found that the median survival of elderly patients admitted to the ICU was 24 months for patients aged 80 to 84 years and 10 months for those aged 85 to 89 years [31,60,61]. Our analysis underscores the importance of early intervention and tailored care strategies to mitigate risks associated with prolonged hospitalization in elderly patients. Implementing comprehensive discharge planning and post-discharge support may improve survival outcomes in this vulnerable population.

Our analysis sought to identify factors associated with nosocomial and community-acquired CDI. We found that older age (*p* = 0.036), presence of watery stools (*p* = 0.033), detection of toxin B (*p* = 0.041), and gastrointestinal comorbidities (*p* = 0.044) were significantly associated with an increased risk of nosocomial CDI. Conversely, vomiting (*p* = 0.001) and cardiovascular comorbidities (*p* = 0.037) were significantly linked to community-acquired CDI. Older age is a well-established risk factor for CDI, with individuals aged 65 or older being particularly susceptible [62]. The association between watery stools and nosocomial CDI underscores the importance of monitoring stool consistency in hospitalized patients. Detection of toxin B suggests a more virulent strain, which may contribute to higher infection rates in healthcare settings. Gastrointestinal comorbidities can disrupt the gut microbiota, increasing vulnerability to CDI [2,63,64,65]. Other factors, including gender, place of residence (rural vs. urban), and most comorbidities (pulmonary, renal, neurological, psychiatric, oncologic, diabetes, obesity, and need for hemodialysis) did not show statistically significant differences between nosocomial and community-acquired infections.

ROC curves were utilized to assess the predictive value of various parameters for recurrence of CDI. The AUC for these parameters was found to be less than 0.700, indicating inadequate predictive value for recurrence. In contrast, age emerged as a significant predictor of mortality in CDI patients. Identification of age as a significant predictor of mortality aligns with the existing literature. Advanced age has been consistently associated with increased mortality risk in various clinical settings [13,49,66,67].

## 5. Conclusions

Our findings emphasize the need for a comprehensive assessment of patients with CDI, considering both comorbid conditions and laboratory parameters. Such an approach can aid in predicting disease severity and tailoring appropriate therapeutic strategies. Understanding these associations is crucial for developing targeted prevention strategies and for early therapeutic intervention. In healthcare settings, heightened vigilance for older patients, those with gastrointestinal issues, or those presenting with watery stools is essential. In the community, awareness of vomiting and cardiovascular conditions as potential risk factors can inform public health initiatives. Further research is needed to explore the mechanisms underlying these associations and to develop tailored interventions for at-risk populations. These findings underscore the importance of considering age in the management and prognostication of CDI patients. While other evaluated parameters may not serve as reliable predictors of recurrence, age remains a critical factor in assessing mortality risk. This highlights the need for tailored interventions and vigilant monitoring of older CDI patients to improve clinical outcomes.

## Figures and Tables

**Table 1:** Correlations between length of hospitalization, symptoms, and epidemiological characteristics.

Epidemiological Characteristics	N (%)	Mean LOS ± SD	Median/Limits	F_ANOVA_ Test
Symptoms				
Watery stools	533 (99.8%)	10.92 ± 7.38	11/1-83	*p* = 0.349
Fever	130 (24.3%)	12.42 ± 8.15	12/1-63	***p* = 0.007**
Vomiting	115 (21.6%)	11.81 ± 10.25	12/1-83	*p* = 0.136
Abdominal pain	394 (73.8%)	10.59 ± 7.55	10/1-83	*p* = 0.098
Demographic data				
Male Female	252 (47.2%) 282 (52.8%)	11.03 ± 6.55 10.79 ± 8.06	11/1-43 11/1-83	*p* = 0.711
Rural Urban	249 (46.6%) 285 (53.4%)	11.13 ± 7.14 10.71 ± 7.58	11/1-63 11/1-83	*p* = 0.508
≤65 years >65 years	301 (56.4%) 233 (43.6%)	9.98 ± 6.27 12.10 ± 8.46	10/1-46 12/1-83	***p* = 0.001**

Abbreviation: LOS—length of stay.

**Table 2:** Correlations between toxin type, comorbidities and laboratory parameters.

Comorbidities	Toxin A + GDH *n* = 89	Toxin B + GDH *n* = 23	Toxin A + B + GDH *n* = 422	Chi^2^ Test
Gastrointestinal, n (%)	7 (7.9%)	6 (26.1%)	98 (23.2%)	***p* = 0.001**
Cardiovascular, n (%)	12 (13.5%)	5 (21.7%)	108 (25.6%)	***p* = 0.036**
Pulmonary, n (%)	5 (5.6%)	2 (8.7%)	42 (10.0%)	*p* = 0.396
Renal, n (%)	4 (4.5%)	6 (26.1%)	42 (10.0%)	***p* = 0.015**
Hemodialysis, n (%)	2 (2.2%)	1 (4.3%)	9 (2.1%)	*p* = 0.823
Neurological, n (%)	9 (10.1%)	7 (30.4%)	54 (12.8%)	*p* = 0.065
Psychiatric, n (%)	5 (5.6%)	2 (8.7%)	21 (5.0%)	*p* = 0.759
Diabetes, n (%)	11 (12.4%)	6 (26.1%)	65 (15.4%)	*p* = 0.301
Oncological, n (%)	18 (20.2%)	5 (21.7%)	50 (11.8%)	*p* = 0.073
Obesities, n (%)	10 (11.2%)	1 (4.3%)	29 (6.9%)	*p* = 0.333
Laboratory parameters				
Inflammatory syndrome, n (%)	82 (92.1%)	22 (95.7%)	374 (88.6%)	*p* = 0.331
Elevated transaminases, n (%)	17 (19.1%)	11 (47.8%)	100 (23.7%)	***p* = 0.025**
Kidney failure, n (%)	27 (30.3%)	10 (43.5%)	114 (27.0%)	*p* = 0.231

**Table 3:** Correlations between toxin type and clinical outcomes in patients with CDI.

Evolution	Toxin A + GDH n = 89	Toxin B + GDH n = 23	Toxin A + B + GDH n = 422	Chi^2^ Square Test
Favorable outcome, n (%)	85 (95.5%)	22 (95.7%)	377 (89.3%)	*p* = 0.097
FMT, n (%)	1 (1.1%)	0 (0.0%)	8 (1.9%)	*p* = 0.582
Recurrence, n (%)	17 (19.1%)	3 (13.0%)	128 (30.3%)	***p* = 0.020**
Mortality, n (%)	4 (4.5%)	1 (4.3%)	45 (10.7%)	*p* = 0.097

Abbreviation: FMT—fecal microbiota transplantation.

**Table 4:** Correlations between treatment and clinical outcomes in patients with CDI.

Evolution	Metronidazole				Chi^2^ Test
	Yes (n = 245)		No (n = 289)		
	n	%	n	%	
Favorable outcome	228	93.1	256	88.6	***p* = 0.050**
FMT	3	1.2	6	2.1	*p* = 0.340
Recurrence	42	17.1	106	36.7	***p* = ** **0.001**
Mortality	17	6.9	33	11.4	***p* = ** **0.050**
**Evolution**	**Vancomycin**				**Chi^2^ Test**
	**Yes (n = 369)**		**No (n = 165)**		
	**n**	**%**	**n**	**%**	
Favorable outcome	326	88.3	158	95.8	***p* = 0.004**
FMT	8	2.2	1	0.6	*p* = 0.178
Recurrence	133	36.0	15	9.1	***p* = 0.001**
Mortality	43	11.7	7	4.2	***p* = 0.004**
**Evolution**	**Vancomycin + Metronidazole**				**Chi^2^ Test**
	**Yes (n = 80)**		**No (n = 454)**		
	**n**	**%**	**n**	**%**	
Favorable outcome	70	87.5	414	91.2	*p* = 0.198
FMT	2	2.5	7	1.5	*p* = 0.401
Recurrence	27	33.8	121	26.7	*p* = 0.121
Mortality	10	12.5	40	8.8	*p* = 0.198

Abbreviation: FMT—fecal microbiota transplantation.

**Table 5:** Clinical outcomes and treatment patterns by CD toxin profile in the study population.

Toxin Type	Recurrence Rate (%)	Treatment Used	Mortality Rate (%)	Favorable Outcome (%)	ESCMID 2014 Recommendations
A + GDH *n* = 89	19.1	Vancomycin 65.1% Metronidazole 51.6% Metronidazole + Vancomycin 17.9%	4.5	95.5	**NON-SEVERE DISEASE** - Metronidazole orally 500 mg × 3 daily for 10 days - Vancomycin orally 125 mg × 4 daily for 10 days - Fidaxomicin orally 200 mg × 2 daily for 10 days **SEVERE DISEASE** - Vancomycin orally 125 mg × 4 daily for 10 days - Fidaxomicin orally 200 mg × 2 daily for 10 days
B + GDH *n* = 23	13.0	Vancomycin 69.5% Metronidazole 39.1% Metronidazole + Vancomycin 8.6%	4.3	95.7	
A + B + GDH *n* = 422	30.3	Vancomycin 70% Metronidazole 45% Metronidazole + Vancomycin 14.6%	10.7	89.3	

**Table 6:** Comparison of patient age and length of hospital stay according to CDI treatment regimen.

Characteristics	Metronidazole (n = 165)	Vancomycin (n = 289)	Vancomycin + Metronidazole (n = 80)	CI 95%	*p*-Value
Age, years (mean ± SD)	55 ± 16.2	61 ± 15.8	61 ± 14.9	−8.9–3.1	<0.001
Hospitalization, days (mean ± SD)	7.9 ± 4.3	12.3 ± 6.8	12.0 ± 6.5	−9.8–2.2	<0.001

Abbreviation: CI—confidence interval.

**Table 7:** Comparison of symptoms and comorbidities in CDI patients by treatment type.

Characteristics	Metronidazole (N = 165)	Vancomycin (N = 289)	Vancomycin + Metronidazole (N = 80)	Chi^2^ Test *p*-Value
Sex, n (%)				
Female	82 (49.7%)	157 (54.3%)	43 (53.8%)	0.624
Male	83 (50.3%)	132 (45.7%)	37 (46.2%)	
Environment, n (%)				
Urban	98 (59.4%)	147 (50.9%)	40 (50.0%)	0.181
Rural	67 (40.6%)	142 (49.1%)	40 (50.0%)	
Clinical symptoms				
Fever	35 (21.2%)	80 (27.7%)	15 (18.8%)	0.099
Vomiting	32 (19.4%)	69 (23.9%)	16 (20.0%)	0.470
Abdominal pain	133 (80.6%)	198 (68.5%)	62 (77.5%)	**0.013**
Comorbidities				
Gastroenterological	35 (21.2%)	59 (20.4%)	17 (21.3%)	0.976
Cardiovascular	24 (14.5%)	91 (31.5%)	11 (13.8%)	**<0.001**
Pulmonary	11 (6.7%)	32 (11.1%)	6 (7.5%)	0.238
Renal	11 (6.7%)	32 (11.1%)	10 (12.5%)	0.170
Hemodialysis	4 (2.4%)	9 (3.1%)	0 (0%)	0.311
Neurological	20 (12.1%)	42 (14.5%)	7 (8.8%)	0.371
Psychiatric	5 (3.0%)	16 (5.5%)	6 (7.5%)	0.197
Diabetes	17 (10.3%)	50 (17.3%)	15 (18.8%)	0.077
Oncological	16 (9.7%)	48 (16.6%)	10 (12.5%)	0.102
Obesity	5 (3.0%)	29 (10.0%)	6 (7.5%)	**0.019**

**Table 8:** Predictors of mortality in patients over 65: logistic regression models.

Logistic Regression Models Deceased, Age > 65 Years Assessed Variables	Odds Ratio (OR)	95% CI	*p* Value
Pulmonary	5.344	3.664–7.795	0.001
Pulmonary	2.858	1.573–5.195	0.001
Renal	2.200	1.194–4.053	0.011
Pulmonary	2.667	1.432–5.006	0.002
Renal	1.798	0.807–4.007	0.151
Diabetes	1.349	0.636–2.860	0.436

Abbreviation: CI—confidence interval.

**Table 9:** AUC values for parameters predicting recurrence.

				Asymptotic 95% Confidence Interval	
Test Result Variable(s)	Area	Std. Error	Asymptotic Sig.	Lower Bound	Upper Bound
Sex	0.527	0.028	0.328	0.473	0.582
Age	0.519	0.029	0.507	0.462	0.576
Environment	0.481	0.028	0.505	0.427	0.536
Nosocomial	0.513	0.028	0.654	0.458	0.567
Watery stools	0.501	0.028	0.963	0.447	0.556
Fever	0.453	0.027	0.093	0.400	0.506
Vomiting	0.490	0.028	0.719	0.435	0.544
Abdominal pain	0.593	0.026	0.001	0.541	0.644
Gastroenterological	0.524	0.028	0.381	0.469	0.580
Cardiovascular	0.488	0.028	0.658	0.433	0.542
Pulmonary	0.516	0.028	0.567	0.461	0.571
Renal	0.484	0.028	0.568	0.430	0.538
Hemodialysis	0.498	0.028	0.957	0.444	0.553
Neurological	0.489	0.028	0.688	0.434	0.543
Psychiatrics	0.487	0.028	0.644	0.433	0.541
Diabetes	0.501	0.028	0.964	0.446	0.556
Oncological	0.494	0.028	0.837	0.440	0.549
Obesity	0.476	0.027	0.395	0.423	0.530
Toxin A	0.516	0.028	0.572	0.462	0.570
Toxin B	0.536	0.027	0.200	0.482	0.589
Toxin A + B	0.552	0.027	0.065	0.499	0.604

Abbreviation: AUC—area under the curve. Test result variable(s): sex, age, environment, nosocomial CDI, watery stools, fever, vomiting, abdominal pain, gastroenterological comorbidities, cardiovascular comorbidities, pulmonary comorbidities, renal comorbidities, hemodialysis, neurological comorbidities, psychiatric comorbidities, diabetes, oncological comorbidities, obesity, toxin A, and toxin B. Toxins A + B had at least one connection between the positive actual state group and the negative actual state group. Statistics may be biased a. under the nonparametric assumption and b. when the null hypothesis true area = 0.5.

**Table 10:** AUC value parameters predicting mortality.

				Asymptotic 95% Confidence Interval	
Test Result Variable(s)	Area	Std. Error	Asymptotic Sig.	Lower Bound	Upper Bound
Sex	0.451	0.043	0.258	0.368	0.535
Age	0.798	0.027	0.000	0.745	0.852
Environment	0.492	0.043	0.860	0.408	0.577
Nosocomial	0.562	0.043	0.147	0.477	0.647
Watery stools	0.501	0.043	0.981	0.417	0.585
Fever	0.476	0.042	0.577	0.394	0.558
Vomiting	0.435	0.039	0.132	0.358	0.513
Abdominal pain	0.457	0.044	0.317	0.371	0.543
Gastroenterological	0.485	0.042	0.720	0.402	0.567
Cardiovascular	0.547	0.044	0.269	0.461	0.634
Pulmonary	0.549	0.045	0.257	0.460	0.637
Renal	0.579	0.046	0.067	0.489	0.669
Hemodialysis	0.510	0.044	0.822	0.424	0.595
Neurological	0.505	0.043	0.909	0.420	0.590
Psychiatrics	0.493	0.042	0.873	0.410	0.576
Diabetes	0.581	0.045	0.060	0.492	0.670
Oncological	0.535	0.044	0.416	0.448	0.622
Obesity	0.459	0.040	0.336	0.380	0.537
Toxin A	0.513	0.042	0.767	0.430	0.595
Toxin B	0.548	0.040	0.265	0.469	0.627
Toxin A + B	0.561	0.040	0.158	0.483	0.638

Abbreviation: AUC—area under the curve. Test result variable(s): sex, age, environment, nosocomial CDI, watery stools, fever, vomiting, abdominal pain, gastroenterological comorbidities, cardiovascular comorbidities, pulmonary comorbidities, renal comorbidities, hemodialysis, neurological comorbidities, psychiatric comorbidities, diabetes, oncological comorbidities, obesity, toxin A, and toxin B. Toxins A + B had at least one connection between the positive actual state group and the negative actual state group. Statistics may be biased a. under the nonparametric assumption and b. when the null hypothesis true area = 0.5.

## Data Availability

Data are contained within the article.

## Supplementary Materials

The following supporting information can be downloaded at https://www.mdpi.com/article/10.3390/microorganisms13061377/s1. Table S1: Univariate analysis of recurrences. Table S2: Estimated risks for nosocomial versus community-acquired CDI.

## Abbreviations

The following abbreviations are used in this manuscript:

AUC	Area under the curve
CD	*Clostridioides difficile*
CDI	*Clostridioides difficile* infection
CI	Confidence interval
ELISA	Enzyme-linked immunoassay
FMT	Fecal microbiota transplantation
GDH	Glutamate dehydrogenase
ICU	Intensive care unit
LOS	Length of stay
ROC	Receiver-operating characteristic curve
