# Supplementary material for: Healthcare-Associated Clostridioides difficile Infection: A Hospital-Based Retrospective Study in North Eastern Romania
Source: Microorganisms. 2025 Jun 13;13(6):1377. doi: 10.3390/microorganisms13061377 (PMC12196210; doi:10.3390/microorganisms13061377)
Supplement: Supplementary file 1 [file microorganisms-13-01377-s001.zip › microorganisms-3640521-supplementary.pdf]

Supplementary Materials

Table S1. Univariate analysis of recurrences.

**Parameters**	**Recurrence (n=148)**	**Chi2 test**
	**Demographics**	
Male	54 (43,2%)	*p* =0.150
Female	84 (56,8%)	
≤ 65 years	77 (52,0%)	*p* =0.124
> 65 years	71 (48,0%)	
Rural	73 (49,3%	*p* =0.249
Urban	75 (50,7%)	
	**Symptoms**	
Watery stools	148 (100%)	*p* =0.723
Fever	26 (17,6%)	***p* =0.014**
Vomiting	30 (20,3%)	*p* =0.353
Abdominal pain	129 (87,2%)	***p* =0.001**
	**Toxins**	
Toxin A	145 (98,0%)	*p* =0.079
Toxin B	131 (88,5%)	***p* =0.029**
Toxins A+B	128 (86,5%)	***p* =0.005**
	**Comorbidities**	
Gastrointestinal, n(%)	36 (24,3%)	*p* =0.130
Cardiovascular, n(%)	32 (21,6%)	*p* =0.315
Pulmonary, n(%)	17 (11,5%)	*p* =0.164
Renal, n(%)	11 (7,4%)	*p* =0.172
Hemodialysis, n(%)	3 (2,0%)	*p* =0.564
Neurological, n(%)	17 (11,5%)	*p* =0.297
Psychiatric, n(%)	5 (3,4%)	*p* =0.164
Diabetes, n(%)	23 (15,5%)	*p* =0.519
Oncological, n(%)	19 (12,8%)	*p* =0.424
Obesities, n(%)	6 (4,1%)	***p* =0.041**

Table S2. Estimated risks for nosocomial versus community-acquired CDI.

**Parameters**	**Nosocomial(N) (n=196)**		**Community-acquired (C) (n=338)**		**Chi² test *p*- Value**	**RR**	**95% CI**
	**n**	%	n	%			
**Demographic**							
**Male**	**97**	49,5	155	45,9	0.236	1,10$_{M}$	0,88-1,37
**Female**	**99**	50,5	183	54,1			
≤ 65 years	100	51,0	201	59,5	**0.036**	1,14$_{>65}$	1,00-1,30
> 65 years	96	49,0	137	40,5			
Rural	91	46,4	158	46,7	0.509	1,01$_{U}$	0,88-1,14
Urban	105	53,6	180	53,3			
**Symptoms**							
Watery stools	196	100,0	337	99,7	**0.033**	1,58$_{N}$	1,48-1,69
Fever	41	20,9	89	26,3	0.096	1,22$_{C}$	0,92-1,61
Vomiting	26	13,3	90	26,6	**0.001**	1,82$_{C}$	1,27-2,60
Abdominal pain	148	75,5	246	72,8	0.279	1,05$_{N}$	0,91-1,21
**Toxins**							
A	186	94,9	325	96,2	0.315	1,19$_{C}$	0,74-1,93
B	171	87,2	274	81,1	**0.041**	1,17$_{N}$	1,01-1,36
A+B	161	82,1	261	77,2	0.107	1,11 $_{N}$	0,86-1,29
**Comorbidities**							
Gastrointestinal	49	25,0	62	18,3	**0.044**	1,17$_{N}$	0,98-1,40
Cardiovascular	37	18,9	88	26,0	**0.037**	1,31$_{C}$	0,98-1,77
Pulmonary	20	10,2	29	8,6	0.316	1,08$_{N}$	0,85-1,37
Renal	20	10,2	32	9,5	0.446	1,03$_{N}$	0,82-1,29
Hemodialysis	2	1,0	10	3,0	0.122	2,30$_{C}$	0,63-7,94
Neurological	26	13,3	44	13,0	0.517	1,01$_{N}$	0,83-1,22
Psychiatric	8	4,1	20	5,9	0.240	1,30$_{C}$	0,72-2,36
Diabetes	32	16,3	50	14,8	0.361	1,05$_{N}$	0,87-1,26
Oncological	29	14,8	44	13,0	0.326	1,06$_{N}$	0,87-1,29
Obesity	15	7,7	25	7,4	0.519	1,01$_{N}$	0,78-1,30

Abbreviation: CI—confidence interval.
